# Knockdown of toll-like receptor 4 signaling pathways ameliorate bone graft rejection in a mouse model of allograft transplantation

**DOI:** 10.1038/srep46050

**Published:** 2017-04-10

**Authors:** Jeng-Long Hsieh, Po-Chuan Shen, Po-Ting Wu, I-Ming Jou, Chao-Liang Wu, Ai-Li Shiau, Chrong-Reen Wang, Hao-Earn Chong, Shu-Han Chuang, Jia-Shiou Peng, Shih-Yao Chen

**Affiliations:** 1 Department of Medical Laboratory Science and Biotechnology, Chung Hwa University of Medical Technology, Tainan, Taiwan; 2 Department of Orthopedics, Tainan Hospital, Ministry of Health and Welfare, Tainan, Taiwan; 3 Department of Orthopedics, College of Medicine, National Cheng Kung University, Tainan, Taiwan; 4 Department of Biochemistry and Molecular Biology, College of Medicine, National Cheng Kung University, Tainan, Taiwan; 5 Department of Microbiology and Immunology, College of Medicine, National Cheng Kung University, Tainan, Taiwan; 6 Department of Internal Medicine, College of Medicine, National Cheng Kung University, Tainan, Taiwan; 7 Department of Pharmacology, Institute for Drug Evaluation Platform, Development Center for Biotechnology, Taipei, Taiwan; 8 Department of Nursing, Chung-Jen Junior College of Nursing, Health Sciences and Management, Chiayi, Taiwan; 9 Department of Internal Medicine, National Cheng Kung University Hospital, College of Medicine, National Cheng Kung University, Tainan, Taiwan

## Abstract

Non-union occurring in structural bone grafting is a major problem in allograft transplantation because of impaired interaction between the host and graft tissue. Activated toll-like receptor (TLR) induces inflammatory cytokines and chemokines and triggers cell-mediated immune responses. The TLR-mediated signal pathway is important for mediating allograft rejection. We evaluated the effects of local knockdown of the TLR4 signaling pathway in a mouse segmental femoral graft model. Allografts were coated with freeze-dried lentiviral vectors that encoded TLR4 and myeloid differentiation primary response gene 88 (MyD88) short-hairpin RNA (shRNA), which were individually transplanted into the mice. They were assessed morphologically, radiographically, and histologically for tissue remodeling. Union occurred in autografted but not in allografted mice at the graft and host junctions after 4 weeks. TLR4 and MyD88 expression was up-regulated in allografted mice. TLR4 and MyD88 shRNAs inhibited TLR4 and MyD88 expression, which led to better union in the grafted sites. More regulatory T-cells in the draining lymph nodes suggested inflammation suppression. Local inhibition of TLR4 and MyD88 might reduce immune responses and ameliorate allograft rejection.

Union after orthopedic reconstruction surgery is essential for evaluating whether the reconstruction was successful. Non-union occurs because of poor interaction between host and graft tissues in bone allograft transplantation^1^. T-cell-mediated immune responses from the recipients against the donor antigens cause graft rejection and poor clinical outcomes^2^,^3^,^4^. Thus, autografts are superior to allografts in bone healing and remodeling because they contain living cells that express self-antigens^5^,^6^. However, chronic pain at the donor sites, size limitations, and complicated procedures are the major drawbacks of autologous bone grafts during transplantation^7^,^8^. To expand the clinical use of allografts, which remain an attractive substitute for autografts, we need a clear and detailed understandings of the mechanisms of immune responses in the bone allograft healing process.

Toll-like receptors (TLRs) are pathogen-associated molecular patterns (PAMPs) receptors that mediate the connection between innate and adaptive immunity^9^,^10^. TLRs sense not only PAMPs but also endogenous ligands like injured tissue and degraded extracellular matrix components, and then they activate downstream signaling that produces cytokines and chemokines, which mediate immune attacks on grafts and cause the grafts to be rejected^11^,^12^. Growing evidence indicates that TLRs are associated with acute allograft rejection. For example, in liver transplant patients, recipients with acute allograft rejection had higher TLR2 and TLR4 expression levels than did recipients with stable transplants and normal liver function^13^,^14^. Increased TLR4 expression was also correlated with endothelial dysfunction in cardiac transplant recipients^15^. Moreover, a minor mismatched skin transplantation mouse model showed that rejection did not occur in the absence of the myeloid differentiation primary response gene 88 (MyD88)^16^, which is a well-known intracellular signal transducer of TLR. Taken together, these observations indicate that TLR-mediated signaling pathway are important for mediating allograft rejection. Inhibiting this pathway provides therapeutic interventions for prolonging graft survival in various transplantation models.

Although the relationship between TLR signaling and transplantation rejection has been established in a variety of parenchymal organs, its role in bone allograft transplantation has not yet been clarified. Therefore, we want to determine whether TLRs and signal transducers are actually involved in the bone allograft healing process. We first established a murine segmental femoral graft model as published previously^17^ and observed that TLR4 and MyD88 were expressed at higher levels in tissue surrounding the grafts from allografted mice compared with autografted and normal mice. Freeze-drying coated allografts in which TLR4 and MyD88 were silenced by lentiviral vectors that expressed short-hairpin (sh) RNA had improved graft repair accompanied by more regulatory T cells (Tregs) in draining lymph nodes (DLNs) of allografted mice.

## Results

### Murine femoral autograft and allograft model

**Figure 1:**
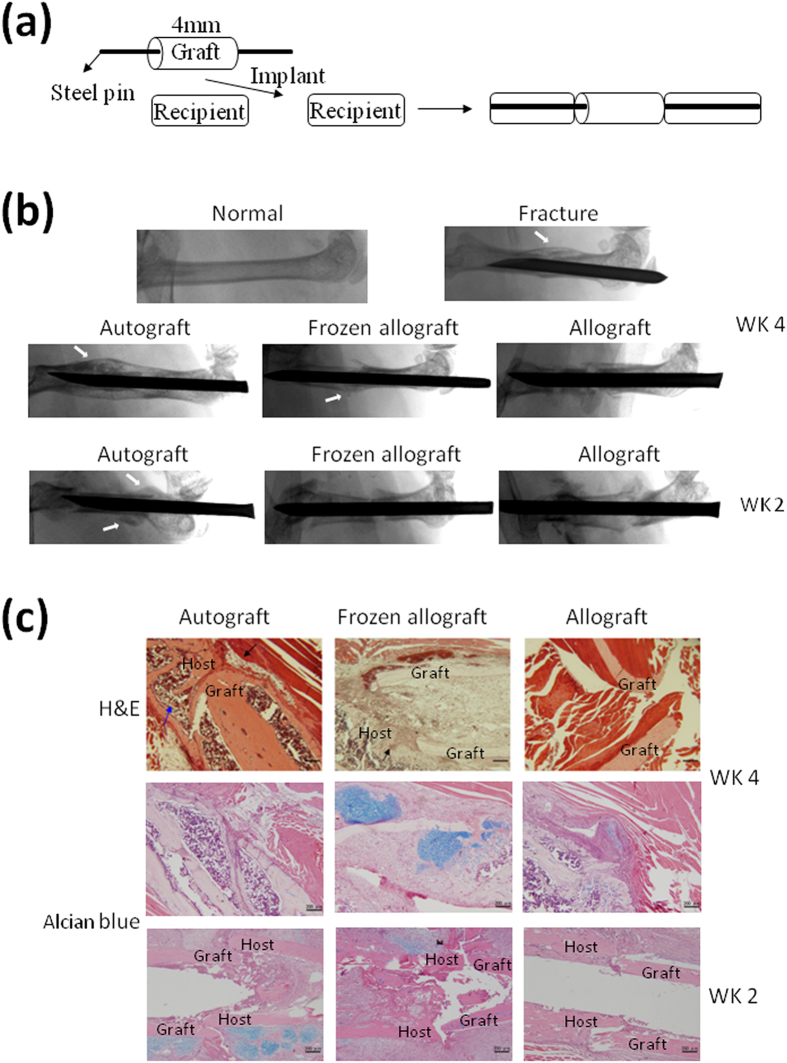
Establishment of a murine femoral allograft model. Mice with femoral bone autografts and allografts were euthanized at 2 (WK 2) and 4 weeks (WK 4) after surgery. (**a**) A schematic figure of the mouse femoral bone transplantation. (**b**) Representative radiographs of autografted, frozen allografted, allografted, fractured, and normal mice at WK 2 and at WK 4 after surgery. White arrows indicate callus formation on the cortical bone surface of the autograft or the frozen allograft at WK 2 or WK 4. (**c**) Representative H&E and alcian blue-stained sections of autografted, frozen allografted, and allografted mice at WK 2 and at WK 4 after surgery. The black arrow indicates periosteal intramembranous bone formation, and the blue arrow indicates new bone-marrow cavity formation. Alcian blue-positive areas can be identified in the frozen allografted mice at WK 4 and autografted mice at WK 2. Scale bars represent 200 μM at 100× magnification.

To determine and clarify the role of TLR4 signaling in bone-graft healing, we set up an *in vivo* murine femoral transplantation model^17^. We first removed a 4-mm section from the middle of the femoral diaphysis of C57BL/6 mice and then placed them back in the original C57BL/6 mice as an autograft or in the allogenic BALB/C mice as an allograft (Fig. 1a). Callus formation started at 2 weeks, and a new bone collar encircled the graft by 4 weeks during autograft healing. In contrast, this did not occur at 2 or 4 weeks during allograft healing, except slightly in the frozen allograft healing at 4 weeks (Fig. 1b). Accordingly, the autograft healing through endochondral ossification at the junctions with intramembranous ossification along the periosteum of the cortex of the graft can be observed after 2 or 4 weeks. Healing was completed at the graft and host junctions, and a new bone marrow cavity had formed after 4 weeks. Notably, neither endochondral nor intramembranous ossification can be identified in the allograft healing (Fig. 1c).

### Increased TLR4 and MyD88 expression during bone allograft healing

**Figure 2:**
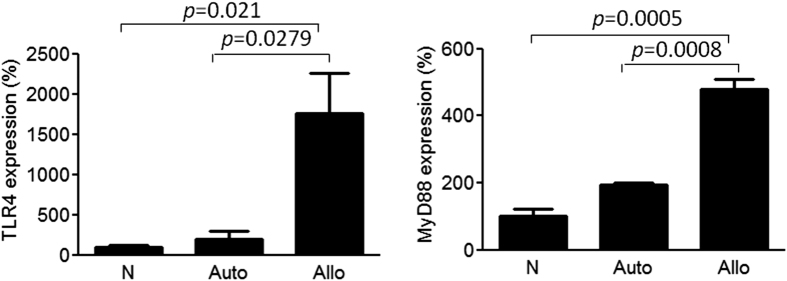
Up-regulation of TLR4 and MyD88 expression in mouse femoral allografts. Mice with femoral bone autografts and allografts were euthanized at 1 or 2 weeks. TLR4 and MyD88 expression in the mouse femoral allograft extracts at 1 week (TLR4) and 2 week (MyD88) after surgery, as determined by quantitative RT-PCR (qRT-PCR). Each value shown represents the mean ± SEM (n = 3).

Because TLR4 has been correlated with various types of acute transplantation rejection^13^,^14^,^15^, we first examined TLR4 expression in the allografted mice. Quantitative analysis using a quantitative reverse transcription polymerase chain reaction (qRT-PCR) showed that TLR4 and MyD88 (the critical signaling transducer) expression had been concomitantly up-regulated in the femoral extracts of the allografted mice (Fig. 2), indicating that TLR4 signaling might play a pivotal role in bone allograft rejection.

### Silencing of TLR4 and MyD88 expression improves bone allograft healing

**Figure 3:**
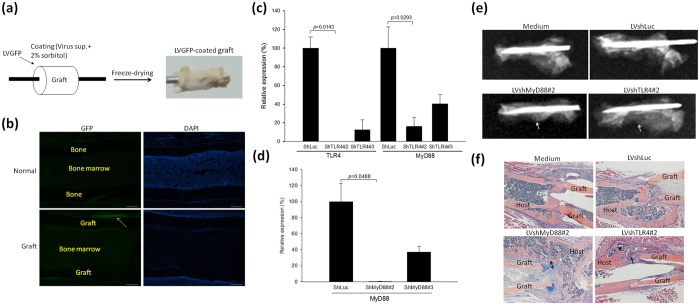
Effects on bone allograft revitalization of silencing TLR4 and MyD88. (**a**) A schematic figure of the lentiviral vectors coated on the graft. The supernatant (Sup.) of the lentivirus that expressed green fluorescence protein (GFP) (LVGFP) was mixed with 2% PBS-buffered sorbitol and coated on the surface of the bone graft by freeze-drying. (**b**) Immunofluorescence staining of GFP (green in an LVGFP-coated allograft). The yellow arrow indicates positivity for GFP expression. Scale bars represent 200 μM in 100× magnification. (**c**) TLR4 or MyD88 expression in MBT2 cells treated with lentiviral vectors that express short-hairpin RNA that targets luciferase (shLuc) or TLR4 (shTLR4), as determined by qRT-PCR. (**d**) MyD88 expression in MBT2 cells treated with lentiviral vectors that express short-hairpin RNA that targets MyD88 (shMyD88), as determined by qRT-PCR. Mice with a femoral bone allograft coated with LVshLuc, LVshMyD88, LVshTLR4, and medium-only were euthanized at 4 weeks. Each value shown represents the mean ± SEM (n = 3). (**e**) Representative radiographs from mice with allografts coated with LVshLuc, LVshMyD88#2, LVshTLR4#2 and medium-only, respectively, at 4 weeks after surgery. There was less bone callus was in mice with allografts coated with LVshLuc and medium-only. The white arrows indicate callus on the cortical surface in mice with allografts coated with LVshTLR4 and LVshMyD88. (**f**) Representative H&E- and alcian blue-stained sections from mice with allografts coated with LVshLuc, LVshMyD88#2, LVTLR4#2, and medium-only, respectively, at 4 weeks after surgery. There was a limited amount of periosteal formation in mice with allografts coated with LVshLuc and medium-only. The arrows indicate the bone- marrow cavity in mice with allografts coated with LVshTLR4 and LVshMyD88. There was new bone collar formation (*) in mice with allografts coated with LVshTLR4. Scale bars represent 500 μM at 40× magnification.

To establish an *in vivo* gene delivery model, we followed previously published methods^18^. Lentiviral vectors carrying green fluorescent protein (LVGFP) was resuspended in a sorbitol solution onto the surface of the graft by freeze-drying, and represented as an “LVGFP-coated graft” (Fig. 3a). We further assessed the transduction efficiency of the LVGFP-coated grafts by transplanting them in allografted mice. Immunoflurescence staining showed GFP signals near the grafted bone (Fig. 3b). To pick up the lentiviral vectors that express shRNA which target TLR4 or MyD88 for the *in vivo* study, MBT2 cells were infected with a lentiviral short-hairpin luciferase reporter gene (LVshLuc), LVshTLR4, or LVshMyD88 and then the stable transfectants were selected. LVshTLR4#2 (shTLR4#2) or LVshTLR4#3 (shTLR4#3) had efficient gene silencing effect on TLR4 or MyD88 (Fig. 3c) and accordingly, LVshMyD88#2 (shMyD88#2) showed gene knockdown effect on MyD88 compared with LVshLuc (shLuc)-transduced cells, as demonstrated by qRT-PCR analysis (Fig. 3d). Therefore, we chose LVshTLR4#2 and LVshMyD88#2 for *in vivo* gene delivery experiments. We coated the allograft with LVshTLR4#2, LVshMyD88#2, and LVshLuc the control vector, and transplanted it in the mice. Radiographic images showed different degree of union in the fracture sites of the four groups. Mice in which LVshTLR4#2- and LVshMyD88#2-coated allografts had been transplanted showed good callus formation (Fig. 3e). Mice in which LVshLuc-coated allografts had been transplanted showed poor callus formation around the fracture site. A histological analysis showed that in mice with LVshTLR4#2- and LVshMyD88#2-coated allografts healed through intramembranous bone formation derived from the cortex of the graft (Fig. 3f). A new marrow space was created at the host and graft junction. After 4 weeks, there was a new bone collar of cortical bone (star mark) in LVshTLR4#2-treated mice. In contrast, mice in which medium-coated allografts had been transplanted showed less periosteal formation on the surface of grafts. Mice in which LVshLuc-coated allografts had been transplanted, the callus had crept from the host side at the junction, and there was a limited amount of periosteal formation on the graft site.

### Regulatory T-cells (Tregs) were generated during bone allograft healing in mice with silenced TLR4 and MyD88

**Figure 4:**
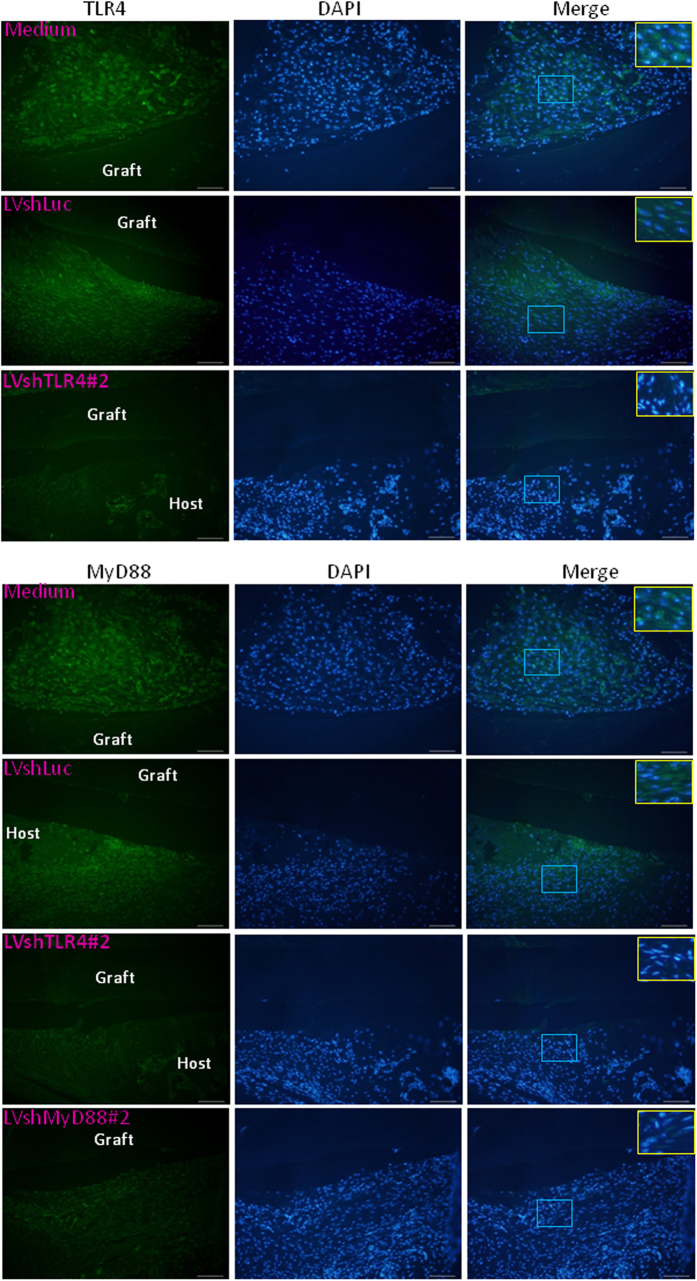
TLR4 and MyD88 expression in LV-coated allografts. Mice with femoral bone allografts coated with LVshLuc, LVshMyD88, LVshTLR4, and medium-only were euthanized at 4 weeks. Immunofluorescence staining of TLR4 and MyD88 (fluorescein isothiocyanate [green]) in the femoral bone allografts coated with LVshLuc, LVshMyD88#2, LVshTLR4#2, and medium-only. Scale bars represent 100 μM at 200× magnification. Higher-magnification views of the merged images shown in the insets correspond to the indicated blue box areas at 200× magnification.

**Figure 5:**
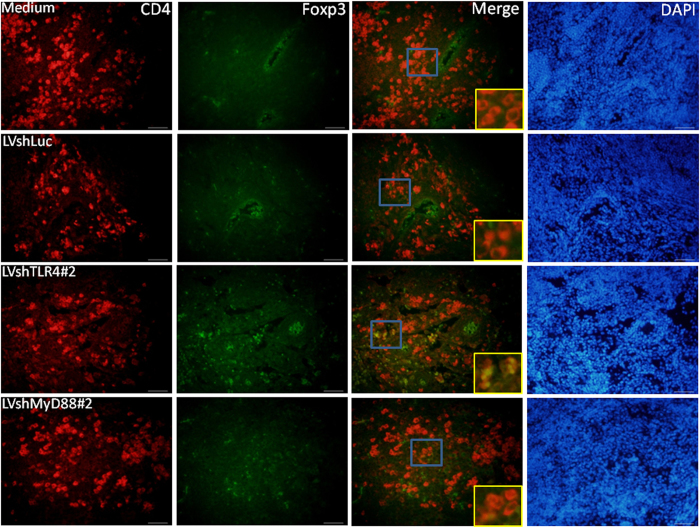
Effects on regulatory T-cells (Tregs) of silencing TLR4 and MyD88 expression. Mice with femoral bone allografts coated with LVshLuc, LVshMyD88, LVshTLR4, and medium-only were euthanized at 4 weeks. Immunofluorescence staining of CD4 (Alexa 594 [red]) and Foxp3 (fluorescein isothiocyanate [green]) in draining lymph nodes from LVshLuc, LVshMyD88#2, LVshTLR4#2, and medium-only-treated mice. Scale bars represent 50 μM at 400× magnification. Higher-magnification views of the merged images shown in the insets correspond to the indicated blue box areas at 400× magnification.

It has been reported that TLR4 signaling is involved in allograft survival through a Treg-dependent mechanism^18^,^19^. We next determined the number of Tregs in the DLNs of mice treated with various lentiviral vectors. The *In vivo* gene knockdown effects of TLR4 and MyD88 were confirmed using immunofluorescence analyses for the tissue surrounding the LVshTLR4 and LVshMyD88-coated allografts, but not in the tissue surrounding the LVshLuc- or medium-coated allografts (Fig. 4). Further quantitative analysis showed lower TLR4 and MyD88 expression in the tissue surrounding the LVshTLR4 and LVshMyD88-coated allografts than in those surrounding the LVshLuc- or medium-coated allografts (For TLR4 expression, LVshTLR4 vs. LVshLuc, 0.38 ± 0.14 vs. 3.09 ± 0.21, *p* < 0.0001, LVshTLR4 vs. medium 0.38 ± 0.14 vs. 2.70 ± 0.88, *p* = 0.0134; For MyD88 expression, LVshTLR4 vs. LVshLuc, 0.26 ± 0.07 vs. 1.54 ± 0.21, *p* = 0.0006, LVshTLR4 vs. medium, 0.26 ± 0.07 vs. 2.17 ± 0.63, *p* = 0.0067, LVshMyD88 vs. LVshLuc, 0.50 ± 0.19 vs. 1.54 ± 0.21, *p* = 0.0173, LVshMD88 vs. medium, 0.50 ± 0.19 vs. 2.17 ± 0.63, *p* = 0.0636). Immunofluorescence staining of DLNs showed that there were more Tregs in mice with LVshTLR4 and LVshMyD88-coated allografts than in those with the control counterparts (Fig. 5). Further quantitative analysis showed higher numbers of Tregs in mice with LVshTLR4 and LVshMyD88-coated allografts than in those with the control counterpart (LVshTLR4 vs. LVshLuc, 70.20 ± 5.45 vs. 27.80 ± 4.39, *p* = 0.0003; LVshMyD88 vs. LVshLuc, 60.25 ± 11.26 vs. 27.80 ± 4.39, *p* = 0.02). Taken together, these data suggest that immune modulation through Tregs might reduce the alloimmune attacks on the grafts by silencing TLR4 and MyD88 expression during bone allograft transplantation.

## Discussion

T-cell-mediated adaptive immunity has long been linked to osteochondral allograft activity^2^,^3^,^4^. The infiltration of osteoblast and osteoclast lineage cells was reduced in allografts when compared to isografts in a murine femoral transplantation model^4^. Nevertheless, the role of innate immunity in the bone transplant healing process has yet to be fully understood. Recently, various transplantation studies have focused on the participation of innate immunity in sharpening the adaptive immune response. Using small interfering RNA (siRNA) to silence the expression of the TLR signal adaptors MyD88 and TRIF prolongs allograft survival in a BALB/C to C57BL6 cardiac transplantation model^18^. In an islet transplantation model^19^, genetic disruption of TLR4 on donor islets had no effect on allograft survival, whereas TLR4 deficiency in recipients led to prolonged graft survival, which was dependent upon the presence of CD4^+^CD25^+^Foxp3^+^ Tregs. These two studies raised that silencing TLR signaling in organ transplantation generated Tregs, which facilitated graft survival.

Tregs are pivotal for maintaining peripheral tolerance by suppressing antigen-specific T-cell proliferation, and inflammatory cytokine production, and by modulating tolerogenic dendritic cell generation through the engagement of cytotoxic T-lymphocyte-associated protein 4 (CTLA4) or CTLA4Ig with B7^20^. Interestingly, a recent study reported the generation of Tregs from the PBMCs of uremic recipients and of healthy donors using *ex vivo* co-stimulation with belatacept (a second-generation CTLA4Ig) during a mixed lymphocyte reaction^21^. It appears to be clinically feasible to use Treg to decrease alloresponsiveness and to limit the need for pharmacologic immunosuppressants during kidney transplantation. Furthermore, targeting TLR4 signaling combined with add-on low-dose rapamycin increased the number of CD4^+^CD25^+^Foxp3 Tregs^22^,^23^, and thus might have synergistic benefits and enable drug minimization in allograft transplantation. Targeting TLR4 signaling should be a potential therapeutic strategy for bone allograft transplantation because Treg cells were generated in DLNs from TLR4- or MyD88-silenced bone allografted mice.

The implantation of solid organs is surgically induced, accompanied by local ischemia-reperfusion injury^24^. Evidence indicates that TLR expression and activation are crucial for the ischemia-reperfusion injury. This type of injury in solid organ transplantation might release putative endogenous ligands that activate antigen presenting cells (APCs) through the engagement with TLRs. APCs stimulated by TLRs will then migrate to DLNs and initiate allogenic T cell-responses against grafts^25^. Studies have reported that TLR4 is capable of sensing a variety of endogenous ligands including high mobility group box protein 1, heat shock proteins, hyaluronan, and fibronectin, which have been implicated in the pathogenesis of acute allograft rejection^26^,^27^. Furthermore, TLR4 mutant mice (C3H/HeJ) had fewer systemic and hepatic inflammatory responses to bilateral femur fracture than did wild-type mice (C3H/HeOuJ)^28^. Tissue damage also occurs during orthopedic reconstruction surgery for spinal fusion, revision hip replacement, and repair of skeletal defects after a tumor resection. These observations indicate that when transplanting solid organs, TLR4 recognizes danger signals released from damaged tissues at the time of transplantation, and that these innate immune signals drive the inflammation and aggravate alloimmune responses. We suggest similar mechanisms during bone allograft transplantation, which require further investigations.

In conclusion, by using a murine femoral allograft model, we are the first to show that innate immunity is crucial in the bone allograft healing process. These findings might contribute to the development of pharmacological therapies targeting the TLR4 signal pathway combining with low-dose immunosuppressants in patients during bone allograft transplantation.

## Methods

### Ethics statement

The Institutional Animal Care and Use Committee of National Cheng Kung University approved the animal experiments. Animal experiments were conducted in accordance with the approved institutional guidelines.

### Murine segmental femoral graft model

Animal experiments were followed by the procedures as published previously^17^. Briefly, 8-week old C57BL/6 mice were intraperitoneally injected with an anesthetic (tiletamine 25 mg/kg and zolazepam 25 mg/kg, Zoletil^®^ 50; Virbac Taiwan Co. Ltd, Taipei City). A 0.7- to 0.8- cm long incision was made to expose the femur and a 0.4 cm mid-diaphyseal segment was removed by using scissors. Bone grafts from C57BL/6 or BALB/C mice were washed with warm phosphate buffered saline (PBS) and transplanted back into C57BL/6 mice, fixed by a 22-gauge metal pin placed through intermedullary marrow cavity, designated autografting or allografting. For frozen allografting, bone grafts from BALB/C mice were harvested, washed with PBS to remove bone marrow cells and rinsed with 70% ethanol and frozen at −70 °C for at least 1 week. Frozen allografts were thawed prior to implantation and then rinsed in saline to remove residual ethanol. Re-implantation of the frozen allografts in to the C57BL/6 mice was performed as described above. Mice were euthanized 2 or 4 weeks after surgery and their grafted femurs were processed for additional examinations.

### Histological and radiographic analysis

After the grafted femurs had been removed from the mice, they were fixed in 10% formalin, decalcified in formic acid, embedded in paraffin, sectioned and stained with hematoxylin and eosin and alcian blue to identify endochondral ossification. Graft healing was evaluated radiographically using an X-ray system.

### Quantitative reverse transcription polymerase chain reaction and immunofluorescent assessments

Total RNA from the femurs was isolated with TRIzol reagents (Invitrogen), and cDNA was synthesized by using Reverse-iT First-strand Synthesis kit (ABgene) for qRT-PCR by SYBR^®^ Premix Ex Taq™ (Takara) with primer pairs specific to TLR4 (forward 5′-ATGGCATGGCTTACACCACC-3′ and reverse 5′-GAGGCCAATTTTGTCTCCACA), MyD88 (forward 5′-TCATGTTCTCCATACCCTTGGT-3′ and reverse 5′-AAACTGCGAGTGGGGTCAG) and GAPDH (forward 5′-GTTGTCTCCTGCGACTTCAAC-3′ and reverse 5′-TTGCTGTAGCCGTATTCATTGTC-3′. The comparative Ct method was used to calculate the relative abundance of TLR4 and MyD88 compared with GAPDH expression. Paraffin-embedded graft and draining inguinal lymph node sections were deparaffinized in xylene, dehydrated in alcohol, treated with proteinase K, washed with H_2_O_2_ in PBS, and stained with antibodies against TLR4 (Santa Cruz), MyD88 (Santa Cruz), GFP (Santa Cruz), CD4 (BD Biosciences), or Foxp3 (eBioscience), followed by Texas red-, FITC- or Alexa 488-conjugated secondary antibodies (BD Biosciences). DAPI was used for nuclear staining. Cells with double stainings of CD4 and Foxp3 were identified and counted in three high-power (400×) field to determine the average percentage of Tregs in each section under fluorescence microscopy. The signal intensity of TLR4 and MyD88 was quantitated using ImageJ software (National Institutes of Health).

### Generation of lentiviral vectors and stable transfectants in which TLR4 or MyD88 was silenced

Mouse TLR4 shRNA-expressing pLKO.1-shTLR4 (TRCN0000065783, TRCN0000065786, and TRCN0000065787) and MyD88 shRNA-expressing pLKO.1-shMyD88 (TRCN0000077233, TRCN0000077234, and TRCN0000077235) and luciferase shRNA-expressing pLKO.1-shLuc (TRCN0000072246) lentiviral plasmids were obtained from the National RNAi Core Facility (Academia Sinica, Taipei, Taiwan). Recombinant lentiviral vectors, LVshTLR4, LVMyD88, LVshLuc, and LVGFP were produced by transient transfection of 293T cells with pLKO.1-shTLR4, pLKO.1-shMyD88, pLKO.1-shLuc, and pWPT respectively, along with the packaging plasmid psPAX2 and the envelope plasmid pMD2G, as previously described^29^. The viral titers were determined by using cell viability assay with A549 cells to calculate relative infection unit (RIU) based on the protocol of the National RNAi Core Facility^29^. On the basis of qRT-PCR analysis, we chose pLKO.1-shTLR4#2 and pLKO.1-shMyD88#2 plasmids (TRCN0000065786 and TRCN0000077234) to generate lentiviral vectors that encoded TLR4 and MyD88 shRNA. To produce TLR4- or MyD88- knocked down stable transfectants, mouse bladder carcinoma MBT2 cells were transduced with LVshTLR4 or LVshMyD88 for 48 h, respectively, in the presence of 8 μg/ml polybrene (Sigma-Aldrich, St. Louis, MO), and the cells were then incubated with puromycin (2 μg/ml) for 2 weeks. Control transfectants were obtained by transduction with LVshLuc, followed by puromycin selection^29^.

### *Ex vivo* gene delivery

We used a previously published the freeze-dry coating method: 1 × 10^6^ RIU of lentiviral vectors in 20 λ of a 1% sorbitol-PBS solution was pipetted onto the cortical surface of the allografts. The grafts were frozen at −80 °C, lyophilized, and stored at −80 °C until they were transplanted^30^.

### Statistical analysis

Data are expressed as mean and SEM. Statistical significance in TLR4 and MyD88 expression levels between different groups was assessed using Student’s *t* test. P values less than 0.05 were considered significant.

## Additional Information

**How to cite this article:** Hsieh, J.-L. *et al*. Knockdown of toll-like receptor 4 signaling pathways ameliorate bone graft rejection in a mouse model of allograft transplantation. *Sci. Rep.* **7**, 46050; doi: 10.1038/srep46050 (2017).

**Publisher's note:** Springer Nature remains neutral with regard to jurisdictional claims in published maps and institutional affiliations.

## References

[b1] Berrey B. H. Jr., Lord C. F., Gebhardt M. C. & Mankin H. J. Fractures of allografts. Frequency, treatment, and end-results. J. Bone Joint Surg. Am. 72, 825–833 (1990). 2365716

[b2] Deijkers R. L. . Human bone allografts can induce T cells with high affinity for donor antigens. J. Bone Joint Surg. Br. 81, 538–544 (1999). 10872380 10.1302/0301-620x.81b3.9265

[b3] VandeVord P. J., Nasser S. & Wooley P. H. Immunological responses to bone soluble proteins in recipients of bone allografts. J. Orthop. Res. 23, 1059–1064 (2005). 15878650 10.1016/j.orthres.2004.12.004

[b4] Fraitzl C. R., Egli R. J., Wingenfeld C., Ganz R., Hofstetter W. & Leunig M. Time course of biological activity in fresh murine osteochondral allografts paralleled to the recipient’s immune response. J. Invest. Surg. 21, 109–117 (2008). 18569430 10.1080/08941930802043540

[b5] Garbuz D. S., Masri B. A. & Czitrom A. A. Biology of allografting. Orthop. Clin. North. Am. 29, 199–204 (1998). 9553565 10.1016/s0030-5898(05)70318-7

[b6] Stevenson S. Biology of bone grafts. Orthop. Clin. North. Am. 30, 543–552 (1999). 10471759 10.1016/s0030-5898(05)70107-3

[b7] Summers B. N. & Eisenstein S. M. Donor site pain from the ilium. A complication of lumbar spine fusion. J. Bone Joint Surg. Br. 71, 677–680 (1989). 2768321 10.1302/0301-620X.71B4.2768321

[b8] Younger E. M. & Chapman M. W. Morbidity at bone graft donor sites. J. Orthop. Trauma 3, 192–195 (1989). 2809818 10.1097/00005131-198909000-00002

[b9] Medzhitov R. Toll-like receptors and innate immunity. Nat. Rev. Immunol. 1, 135–145 (2001). 11905821 10.1038/35100529

[b10] Schnare M. . Toll-like receptors control activation of adaptive immune responses. Nat. Immunol. 2, 947–950 (2001). 11547333 10.1038/ni712

[b11] Alegre M. L., Goldstein D. R. & Chong A. S. Toll-like receptor signaling in transplantation. Curr. Opin. Organ Transplant. 13, 358–365 (2008). 18685330 10.1097/MOT.0b013e3283061149 PMC2605274

[b12] Goldstein D. R. Toll like receptors and acute allograft rejection. Transpl. Immunol. 17, 11–15 (2006). 17157206 10.1016/j.trim.2006.09.012

[b13] Deng J. F. . The role of toll-like receptors 2 and 4 in acute allograft rejection after liver transplantation. Transplant Proc. 39, 3222–3224 (2007). 18089358 10.1016/j.transproceed.2007.02.102

[b14] Testro A. G. . Acute allograft rejection in human liver transplant recipients is associated with signaling through toll-like receptor 4. J. Gastroenterol. Hepatol. 26, 155–163 (2011). 21175809 10.1111/j.1440-1746.2010.06324.x

[b15] Methe H., Zimmer E., Grimm C., Nabauer M. & Koglin J. Evidence for a role of toll-like receptor 4 in development of chronic allograft rejection after cardiac transplantation. Transplantation 78, 1324–1331 (2004). 15548971 10.1097/01.tp.0000137930.40597.03

[b16] Goldstein D. R., Tesar B. M., Akira S. & Lakkis F. G. Critical role of the Toll-like receptor signal adaptor protein MyD88 in acute allograft rejection. J. Clin. Invest. 111, 1571–1578 (2003). 12750407 10.1172/JCI17573 PMC155048

[b17] Tiyapatanaputi P. . A novel murine segmental femoral graft model. J. Orthop. Res. 22, 1254–1260 (2004). 15475206 10.1016/j.orthres.2004.03.017

[b18] Zhang X. . Induction of alloimmune tolerance in heart transplantation through gene silencing of TLR adaptors. Am. J. Transplant. 12, 2675–2688 (2012). 22823145 10.1111/j.1600-6143.2012.04196.x

[b19] Zhang N. . Inhibition of TLR4 signaling prolongs Treg-dependent murine islet allograft survival. Immunol. Lett. 127, 119–125 (2010). 19879295 10.1016/j.imlet.2009.10.004 PMC2808455

[b20] Mulley W. R. & Nikolic-Paterson D. J. Indoleamine 2,3-dioxygenase in transplantation. Nephrology (Carlton) 13, 204–211 (2008). 18221253 10.1111/j.1440-1797.2007.00921.x

[b21] Guinan E. C. . *Ex vivo* costimulatory blockade to generate regulatory T cells from individuals awaiting kidney transplantation. Am. J. Transplant. Jan 21 [Epub ahead of print] (2016). 10.1111/ajt.13725 26790369

[b22] Battaglia M., Stabilini A. & Roncarolo M. G. Rapamycin selectively expands CD4 + CD25 + FoxP3 + regulatory T cells. Blood 105, 4743–4748 (2005). 15746082 10.1182/blood-2004-10-3932

[b23] Qu Y. . The effect of immunosuppressive drug rapamycin on regulatory CD4 + CD25 + Foxp3 + T cells in mice. Transpl. Immunol. 17, 153–161 (2007). 17331841 10.1016/j.trim.2007.01.002

[b24] Boros P. & Bromberg J. S. New cellular and molecular immune pathways in ischemia/reperfusion injury. Am. J. Transplant. 6, 652–658 (2006). 16539620 10.1111/j.1600-6143.2005.01228.x

[b25] Alegre M. L., Goldstein D. R. & Chong A. S. Toll-like receptor signaling in transplantation. Curr. Opin. Organ. Transplant. 13, 358–365 (2008). 18685330 10.1097/MOT.0b013e3283061149 PMC2605274

[b26] Huang Y. . Extracellular hmgb1 functions as an innate immune-mediator implicated in murine cardiac allograft acute rejection. Am. J. Transplant. 7, 799–808 (2007). 17331117 10.1111/j.1600-6143.2007.01734.x

[b27] Termeer C. . Oligosaccharides of Hyaluronan activate dendritic cells via toll-like receptor 4. J. Exp. Med. 195, 99–111 (2002). 11781369 10.1084/jem.20001858 PMC2196009

[b28] Levy R. M. . Systemic inflammation and remote organ damage following bilateral femur fracture requires Toll-like receptor 4. Am. J. Physiol. Regul. Integr. Comp. Physiol. 291, R970–976 (2006). 16675630 10.1152/ajpregu.00793.2005

[b29] Chen S. Y. . Transcription factor snail regulates tumor necrosis factor α-mediated synovial fibroblast activation in the rheumatoid joint. Arthritis Rheumatol. 67, 39–50 (2015). 25303734 10.1002/art.38899

[b30] Ito H. . Remodeling of cortical bone allografts mediated by adherent rAAV-RANKL and VEGF gene therapy. Nat. Med. 11, 291–297 (2005). 15711561 10.1038/nm1190 PMC1364464

